# Blending Insights from Implementation Science and the Social Sciences to Mitigate Inequities in Screening for Hereditary Cancer Syndromes

**DOI:** 10.3390/ijerph16203899

**Published:** 2019-10-15

**Authors:** Laura Senier, Colleen M. McBride, Alex T. Ramsey, Vence L. Bonham, David A. Chambers

**Affiliations:** 1 Department of Sociology & Anthropology, Northeastern University, 360 Huntington Avenue, Boston, MA 02115, USA; 2 Department of Health Sciences, Northeastern University, 360 Huntington Avenue, Boston, MA 02115, USA; 3 Department of Behavioral Sciences & Health Education, Rollins School of Public Health, Emory University, 1518 Clifton Road NE, Atlanta, GA 30322, USA; colleen.marie.mcbride@emory.edu; 4 Department of Psychiatry, Washington University School of Medicine, 660 South Euclid Avenue, St. Louis, MO 63110, USA; aramsey@wustl.edu; 5 Social and Behavioral Research Branch, National Human Genome Research Institute, National Institutes of Health, 31 Center Drive, Bethesda, MD 20892, USA; bonhamv@mail.nih.gov; 6 Division of Cancer Control and Population Sciences, National Cancer Institute, 9609 Medical Center Drive, Rockville, MD 20850, USA; dchamber@mail.nih.gov

**Keywords:** consolidated framework for implementation research, genomics, public health, health inequities

## Abstract

Genomic screening to identify people at high risk for adult-onset hereditary conditions has potential to improve population health. However, if not equitably accessible, genomics-informed screening programs will exacerbate existing health inequities or give rise to new ones. To realize the disease prevention potential of these screening tools, we need strategies to broaden their reach. We propose a conceptual framework that merges insights from implementation science and sociological research on health inequities. Our framework does three things: first, it broadens the arenas of action beyond those typically addressed in implementation science frameworks; second, it argues for recruiting more diverse partners to share the work of implementation and dissemination; and third, it shows how implementation activities can be coordinated more effectively among those partners. We use screening for hereditary breast and ovarian cancers (HBOC) as a case to illustrate how this enhanced framework could guide implementation science and distribute the benefits of genomic medicine more equitably. Although our example is specific to genomics, this approach is more broadly applicable to the field of implementation science. Coordinated action among multiple stakeholders could translate a host of new technologies from the bench to the trench without creating new inequities or exacerbating existing ones.

## 1. Introduction

Genomic screening to identify people at high risk for adult-onset hereditary conditions has tremendous potential to improve population health [1,2,3,4,5]. However, critics have warned that genomics-informed screening programs might exacerbate existing health inequities or give rise to new ones, if they are not fairly available to all patients who might benefit from them [6]. Moreover, the US healthcare system is struggling to implement these genomic applications into routine clinical and public health practice, even when they are backed by expert panel recommendations. To overcome these translational barriers and achieve the disease prevention potential of these screening tools, we need to monitor outcomes of current programs and establish new strategies to broaden their reach [1,7]. Accordingly, federal agencies have made funding available to promote evidence-based practice and to spur implementation science that will identify and eliminate barriers to broaden population-level genomic screening. However, much of this work in genomics has been descriptive, interventions to improve uptake have rarely been grounded in theories from implementation science or social and behavioral sciences, and very little of this work has empirically evaluated whether implementation of genomic screening is actually widening health inequities [8]. 

While reviews have identified more than 60 conceptual frameworks from implementation science that identify influences on implementation at individual, organizational, and system levels [9,10], few of them have been utilized to optimize uptake of genomic screening. Similarly, research in the social and behavioral sciences has yielded multilevel frameworks that explicate the patient-, provider-, and community-level factors that constrain or enable healthcare delivery, but these frameworks have not been utilized to support genomic screening interventions and implementation [11,12,13,14,15]. Consequently, remarkably little of the work done thus far to use genomic screening to identify people at high risk for hereditary, adult-onset conditions has led to the development of recruitment, engagement, education, consenting, or intervention approaches that will improve the reach of genomic medicine while mitigating or forestalling inequities in access to genomic applications. 

In this paper, we argue not only for a more disciplined use of theory in designing, implementing, and evaluating screening programs that will integrate genomic applications, but also that the conceptual frameworks currently guiding implementation science could be integrated with insights from the social and behavioral sciences, to better detect and prevent the emergence of health inequities. We urge researchers who are working to overcome barriers in research translation to not only leverage the tools and insights of implementation science, but to also actively embrace the question, “If the intervention being tested in my study has the hypothesized effect, will health inequities increase or decrease?” This question forces us to be mindful of the choices we make in crafting our research questions and selecting health systems and communities where we locate our studies, because these choices can impact equity. If, for example, researchers choose to work only within well-resourced, high-performing health systems, any additional improvements in health will increase the magnitude of disparities with other settings. Similarly, if we assume that implementation should always occur, without considering whether there is a mismatch between the intervention and the population or setting where it is intended to be implemented, our studies may make practice in those settings even harder. 

We propose a framework that draws upon the Consolidated Framework for Implementation Research (CFIR) and selected theories from sociological research on health inequities, most notably fundamental cause theory, and show how this framework could be applied to mitigate inequities in the translation and implementation of genomic applications. Our adapted framework does three things. First, it broadens the arenas of action beyond those typically addressed in implementation science; second, it proposes recruitment of more diverse partners to share in the work of implementation and dissemination; and third, it suggests how implementation activities can be coordinated among those partners in a more orchestrated fashion. Specifically, the framework expands the areas the CFIR includes in the “outer setting” and makes them more endogenous to the investigation, to address the social, political, and economic barriers that patients may encounter when attempting to access healthcare. We also show how stakeholders who have been underemphasized within the CFIR (e.g., patient advocacy groups, public health officials, and policymakers) can nudge implementation science in new directions toward identifying and mitigating inequities, especially by taking action at policy levels outside the clinic. Throughout, we use screening for hereditary breast and ovarian cancers (HBOC) as a case study to illustrate how this framework may be fruitful for guiding the development and evaluation of interventions that will distribute the benefits of genomic medicine more equitably. 

## 2. Clarifying Terminology: Theorizing and Defining Health Inequities 

Scholars from many different disciplines use the terms “health disparities,” “healthcare disparities,” “health inequalities,” and “health inequities,” often interchangeably. For example, some genomic researchers use the term “health disparities” when describing differential health outcomes (e.g., prostate cancer, breast cancer), and in describing population-level differences in genetic variation and disease [16,17]. Others refer to health disparities when referring to misclassification of disease variants and resulting misdiagnoses of patients based upon lack of diverse ancestral data in genetic-testing laboratory clinical databases [18]. This interpretation of disparities typically aims to inform the development of new treatments and diagnosis for diseases that differentially affect certain ancestral populations. Conversely, some public health researchers and social scientists use the term “health disparities” to refer to differences in health outcomes that stem from social inequalities [19]. Similarly, the term “racial and ethnic healthcare disparities,” characterizes differences in treatment provided to members of different racial or ethnic groups that is not justified by the underlying health conditions or treatment preferences of those patients [20]. Research from this perspective often seeks to inform policies to make access to health services more equitable. 

We use the term “health inequities” in this paper to describe “a subset of health and healthcare disparities that stem from a chain of events that confer advantages to some groups over others, and as such, are modifiable and ethically unfair [21].” In the case of HBOC screening, for example, some of the differences in health outcomes observed among population subgroups constitute health disparities (e.g., the higher prevalence of *BRCA1/2* variants among Ashkenazi Jewish women and triple-negative breast cancer among African-American women) [17,22], whereas others result from the interplay of social forces that make some groups more vulnerable than others (e.g., African-American women not being offered testing, being unable to pay for testing because of poverty or insurance status, or cultural barriers that prevent providers from communicating risks and benefits to this group) [23,24,25,26,27]. We believe this latter group of differences in health outcomes constitutes true health inequities and will require coordinated stakeholder action to rectify.

## 3. Hybridizing Implementation Science and Sociological Research

Implementation science proffers numerous frameworks that account for multilevel influences that affect delivery of clinical care, while also examining the characteristics of an intervention that hinder its adoption or sustainability [9,28]. These frameworks typically articulate implementation as a multi-staged process, beginning with exploration and planning phases, followed by integration of an intervention in a defined clinical or community setting, and then a period of evaluation, routinization, and institutionalization. These frameworks recognize important influences across these different stages, frequently identifying local barriers and facilitators to adoption and implementation (e.g., organizational resources, staff knowledge and attitudes, community culture), as well as broader environmental influences that drive health and healthcare (e.g., economy, societal norms, policy). Few of these frameworks, however, examine how implementation affects health inequities, and they fail to account explicitly for the broader social, political, and economic forces that hinder access to care. This important omission may have the unintended consequences of spurring research that concentrates implementation on settings and populations that have greater resources, or implicitly regards settings and populations as having equal social footing, thus inadvertently entrenching inequities in provision of care. 

Similarly, social and behavioral science scholars have developed conceptual frameworks to explicate social, political, and economic forces that drive inequitable distribution and variable quality of health services and simultaneously constrain the choices of people who live in disadvantage [11,12,13,14,15]. Some frameworks are grounded in the experiences of an individual person or patient, and begin with an inventory of biological conditions, genetic factors, and health habits that contribute to the person’s health, but then also account for neighborhood, clinic, and social factors that shape those health habits and behaviors. Other frameworks take a more top-down approach, and seek to understand how macro-level forces such as globalization, urbanization, immigration, and deindustrialization create healthy or unhealthy neighborhoods or communities, and then seek to understand the effects of these macro-level forces on populations [13,29,30,31]. To our knowledge, however, none of these frameworks have yet been applied to implementing or evaluating genetic screening programs for their impact on health inequities. We aim to blend insights from these two currents of theory to inform implementation of evidence-based genomic recommendations in ways that will enhance population health equitably. 

Sociological theories of health inequities view demographic characteristics such as race, sex, gender, and socioeconomic status as proxies for social processes or historical events that have conferred wealth and advantage on some people, while marginalizing or exploiting others. Sociologists regard these social attributes as *fundamental causes of disease* that represent social processes that structure and constrain opportunities of everyday living [13]. Viewed in this way, we see that a variable such as income or socioeconomic status is not just an attribute of an individual, but signifies an individual’s ability to purchase healthcare or pursue a lifestyle that allows them to avoid health risks. Furthermore, fundamental cause theory holds that the mechanisms that influence disease outcomes operate at multiple levels in complex interplay, thus allowing for “advantage to accrue change from place to place and from time to time [13,32].” New pathways for inequities can emerge over time, even when interventions temporarily succeed in mitigating them. Because implementation science recognizes the frequent emergence of new implementation barriers [33], we believe that it is compatible with fundamental cause’s focus on fluid and shifting forces driving health inequities.

## 4. Potential for Implementation of Genomic Applications to Promote or Mitigate Health Inequities: The Case of HBOC

We use HBOC screening as our example, primarily because health inequities in breast cancer screening, treatment, and prevention are already rife within the U.S. healthcare system. In this context, optimal dissemination of HBOC screening will inevitably face formidable challenges—and a moral imperative—to implement screening in ways that will not exacerbate or entrench existing health inequities. Moreover, HBOC screening has been endorsed by numerous expert panels since 2005 [4,5,7], meaning that it should (at least in theory) be the standard of care, and yet we see differences in uptake among different groups. 

Passage of the Affordable Care Act (ACA) in 2010 was widely expected to make it easier for people to access healthcare, but progress toward that ideal has been fraught with political and logistical challenges. First, the scope of the ACA was limited to encouraging expansion of insurance coverage for primary and preventive care. The ACA reformed regulations on private insurance policies covering many Americans and expanded subsidies for public insurance programs for those not covered in the private markets [34]. Second, the main criteria for determining which screening and preventive services should be covered was limited to services that had been approved by the US Preventive Services Task Force (USPSTF). To date, the USPSTF has recommended population-level screening for only one hereditary condition—HBOC [3,4,5]. Third, a judicial ruling from the US Supreme Court in 2012 gave states the option (rather than the duty) of expanding public insurance programs to include poor patients. As a result, there are wide disparities in coverage in different regions of the US [35,36], even for services that should be part of the recommended package of basic and essential services, such as HBOC screening. Moreover, the focus on financing primary care has still left many Americans without adequate coverage for specialty healthcare services such as genetics. This saga illustrates how the quest for near-universal payment for any healthcare service can still fail to address the cultural, institutional, and systemic barriers that impede research translation. 

Although HBOC accounts for only 10% of cases of breast cancer in the US annually, women who carry *BRCA1/2* variants have a cumulative lifetime risk of up to 80% compared to typical women, whose cumulative lifetime risk is only 12% [37]. Moreover, higher rates of early disease onset and death among these high-risk individuals and their family members carry significant social costs (e.g., healthcare expenditures, years of life lost). At-risk women can take preventive actions including earlier surveillance, prophylactic mastectomy, and pharmacologic prevention [38,39,40,41]. Finally, identifying at-risk women and referring them for genetic consultation is relatively easy, given the availability of well-validated and inexpensive family history tools; under $100 for families and less than $5 per individual at risk [42]. 

In 2005, the U.S. Preventive Services Task Force (USPSTF) endorsed population-level HBOC screening, especially for women with a strong family history of the disease [3,4,5,43]. Yet, to date, uptake of HBOC screening has been uneven, with most programs identifying individuals in specialty care settings or relying on tumor registries, domains in which well-educated whites and those who already have cancer are overrepresented [44,45]. Efforts to implement HBOC screening in primary-care, community-based settings have found that poor women and women of color are less likely to be screened than their white and wealthier peers [46,47,48,49]. 

To be sure, the prospect of population-level screening for genetic variants raises a host of ethical questions about potential harms or unintended consequences. The current situation in the US, however, shows marked health inequities in screening, treatment, and prevention for typical breast cancers, reflecting the influence of social conditions as fundamental causes of disease [50]. This shows that we need more attention to implementing treatments for all kinds of cancers, and that implementation scientists could play an important role in expanding services if they are attuned to health inequities. African-American and Hispanic women are less likely to get breast cancer than white women, but more likely to develop the disease at a younger age; develop aggressive, hormone receptor-negative cancers; be diagnosed at a later stage of the disease; and die at an earlier age [51,52]. These differences in breast cancer mortality have been attributed to a “perfect storm” of biology, behavior, and service access [51,53]. Disparate outcomes in breast cancer morbidity and mortality reflect social inequities that force poor women and women of color to live in poverty, to make choices about their educational and occupational trajectories that condition their reproductive decision making, and expose them to environmental carcinogens and high levels of toxic stress [54]. These living and working conditions also shape the options women have for employment, insurance coverage, and access to high-quality healthcare [54,55]. These inequities are so thoroughly entrenched in delivering care for typical breast cancers that it is even more imperative that we carefully consider what might be done to screen for HBOC, so that we avoid replicating these patterns of inequities. Moving forward with recommendations for population-level genetic screening will undoubtedly require input from ethicists to ensure that everyone can benefit and that certain groups will not be disproportionately impacted, but implementation scientists should also consider equity as an outcome of their work. 

We already know that efforts to disseminate population-level HBOC screening have shown limited reach and slower uptake among underserved and minority groups; a pattern that is all too familiar in the diffusion of most innovations [44,45]. For example, African-American women with a family history of breast cancer are significantly less likely than white women to undergo genetic counseling, even after adjusting for probability of mutation, socioeconomic status, and physician recommendation [24,25,27,47]. African-American women are also less likely to be aware of HBOC screening than white women [47,56,57]. While some studies have suggested that these differential rates of screening reflect patient preferences and values [58], it is also likely that they stem from social, political, and economic forces that structure access to care and quality of care in the US [51,54].

Studies have also shown differential utilization of HBOC screening among other racial and ethnic minorities. Vietnamese immigrants, for example, are less likely to seek information on cancer and less likely to be referred to electronic, print, and interpersonal sources of advice on cancer; and Spanish-speaking Latinas are much less likely to discuss genetic testing with a healthcare provider than white women [56,59]. Poor women face similar barriers; a 2002 study showed that as many as one quarter of women at risk for HBOC declined screening because of concerns about the cost [60]. Finally, genetic services are usually concentrated in cities or at academic medical centers, making it extremely difficult for rural populations to access genetic counseling, screening, and treatment [61,62]. The regional maldistribution of health services is a major barrier driving rural-urban inequities for many types of healthcare services.

The forces driving inequities in breast cancer screening, treatment, and prevention are pervasive; reflect social, political, and economic conditions operating in multiple arenas of action; are historically contingent; and respond fluidly to changing political or economic conditions. Hence, we consider this “perfect storm” of biology, behavior, and service access a good opportunity for demonstrating how implementation science could be adapted to keep social inequities and fundamental causes in mind.

## 5. Needed Components in an Integrated, Multilevel Framework for Implementation Science and Equity

We begin our approach to intervening in health inequities in genomic medicine with the Consolidated Framework for Implementation Research (CFIR), one of the most frequently used frameworks in the field of implementation science [28]. We propose adding a broader array of social, political, and economic forces that inhibit patients’ treatment seeking and providers’ service delivery to the CFIR’s definition of “outer setting” factors. Moreover, in line with CFIR’s focus on the “fit” between the intervention, the clinic’s “inner setting,” and the adaptable periphery, we bring attention to a broader array of organizational cultures, operational constraints, and the broader context in which patients live, work, and play. In what follows, we show that the CFIR and fundamental cause theory frameworks are highly compatible, and can encourage implementation efforts that are sensitive and responsive to threats of health inequities, whether they arise from intra-clinic or external factors. 

**Figure 1:**
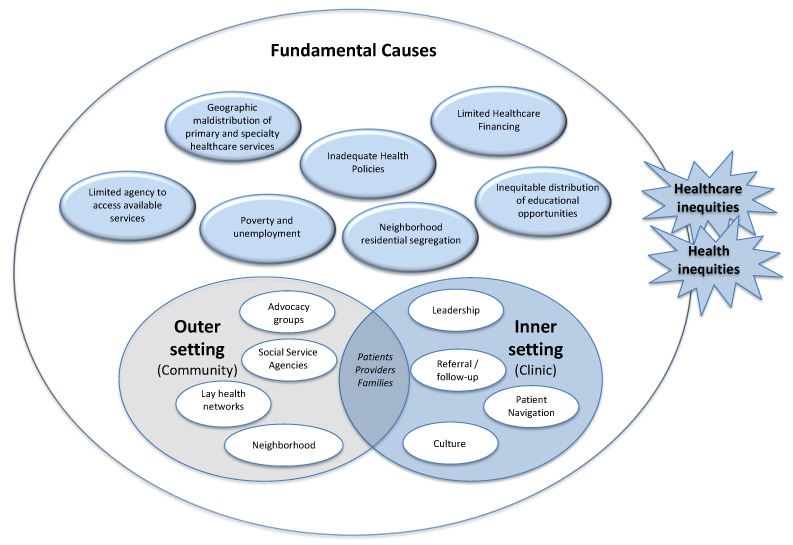
Arenas of action. This figure shows the major arenas of action typically included in implementation science frameworks such as the CFIR, but we expand the outer setting to include a broader array of political, social, and economic forces—fundamental causes that affect the way healthcare delivery unfolds. The figure shows examples of each setting that could facilitate healthcare seeking, or become barriers in access to care that might exacerbate health inequities or give rise to new ones.

In Figure 1, we identify the major arenas of action that should be addressed in implementation science. Health inequities will almost certainly arise in any of these settings. We include the settings the CFIR has traditionally examined, i.e., the inner setting (e.g., patient-, provider-, and family-level factors, and clinic-level organization) and the “outer setting” (e.g., area and community level factors). However, we expand the area the CFIR calls the “outer setting” to specifically address the broader political, social, and economic contexts where healthcare delivery unfolds. Anticipating inequities in access and planning for implementation to mitigate those barriers would need would need to address practical barriers that might prevent patients from accessing care, such as transportation, childcare, or translation services. However, historical legacies of neighborhood residential segregation have concentrated poverty, unemployment, and low educational attainment in communities where many racial and ethnic minorities live, making it difficult for them to get to a healthcare facility that provides genetic services, or making them suspicious and mistrustful of the healthcare system as a whole. Preventing inequities resulting from fundamental causes that drive the geographical maldistribution of healthcare services will likely require policy-level interventions beyond the clinic, and the cooperation of partners who are not typically involved in implementation science work. 

**Figure 2:**
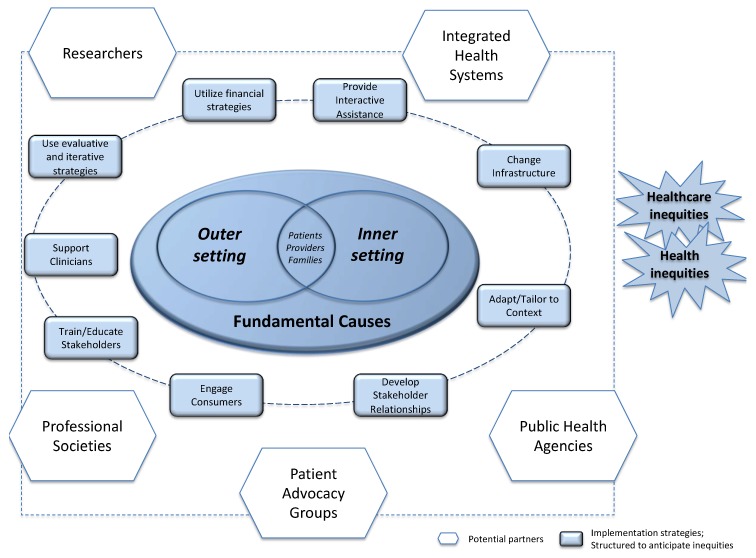
Framework merging implementation science arenas, stakeholders, and strategies with insights from social scientific research on health inequities. Like other implementation science frameworks, we place patients, providers, and families at the center of the action, and show that their healthcare seeking behaviors are affected by—and potentially limited by—factors in the inner clinic setting and outer clinic setting. We also show, however, that fundamental causes of disease (e.g., poverty, racism, structural inequalities) exert their influence. The heavy dashed line connects the nine major categories of implementation science strategies identified by the ERIC study [63,64]. The lighter dashed line connects the stakeholders who should play a role in dissemination and implementation efforts. Any of the nine categories of implementation science strategies identified by the ERIC study could be used by any of the stakeholders; indeed, an effective implementation may require the involvement of multiple stakeholders, each bringing their skills to the project.

In Figure 2, we show how patient advocacy groups, academic researchers, public health officials, health professional societies, and policymakers can design interventions so that they extend further into the outer setting and address broader, fundamental causes. While there are many ways of characterizing implementation science work, our figure shows the nine categories identified in the Expert Recommendations for Implementing Change (ERIC) study [63,64]. For example, the category of “Change Infrastructure” strategies might be implemented by health system executives (e.g., change record systems, making it easier to flag women with a family history of breast cancer for referral to genetics) or by professional associations (e.g., create or change credentialing and licensure standards, to promote state-level licensure of genetic counselors, making it easier for health systems to bill for their time). As another example, the category of “Adapt and Tailor to Context,” includes “use data warehousing techniques” as a strategy. Executives in integrated health systems certainly have access to patient data that provide insights into the user patterns of their systems, but what they may not realize is that public health officials also have data sources—such as state cancer registries—at their disposal, and that pooling data could yield greater insights about how to identify patients who would benefit from screening. 

Here is where our model shows its greatest utility—in highlighting the importance of collaborations with multiple stakeholders, including those not conventionally included in the CFIR. Partnerships that span service settings and draw in unusual partners can advance implementation science and basic research in multiple ways. For example, several state public health agencies have been working to integrate genomics into their chronic disease prevention programs over the past decade, with modest funding from the Centers for Disease Control and Prevention (CDC) [65,66,67,68]. Academic researchers could advance the field of public health genomics greatly by partnering with these agencies to evaluate their work and demonstrate the impact and the reach of these projects. As another example, boosters of telemedicine initiatives tout their potential to improve access to care, either by providing distance education for primary care providers in rural areas, or by supporting remote consultation for patients. However, telemedicine could also drive health inequities if programs are not implemented thoughtfully to reach people in greatest need, and if the services are not covered by insurance. A long-running project that educates primary care providers in rural areas (Project Extension for Community Healthcare Outcomes (ECHO)) about chronic health conditions has been shown to increase provider confidence in treating patients with hepatitis C, asthma, and uncontrolled diabetes, and a few studies have documented modest improvements in patient health outcomes [69]. Although Project ECHO has not yet been evaluated for its potential to broaden access to clinical genetics services, it holds some promise for increasing reach of these innovations. However, a survey of genetics providers in seven states and the District of Columbia found that insurance covered only about 47% of telegenetics services [70], suggesting that telemedicine is not the panacea that some have expected it to be. For these reasons, our model illustrates the importance of drawing together multiple parties when “changing service sites” or “providing interactive assistance.” In the examples that follow, we illustrate how collaborations among stakeholders across arenas may provide a more complete picture of who is and is not being served, and allow for more creativity in designing programs to reach those groups. 

Our expanded CFIR framework is consonant with more recent efforts to bring an action-oriented lens to the CFIR, by using the constructs to diagnose implementation barriers in specific contexts [71,72,73]. To illustrate the potential of an expanded CFIR framework, we present a few examples of actual programs that have been developed by the sort of multi-stakeholder partnerships we are describing, and which have succeeded in mitigating barriers in access to HBOC screening. Our examples cover different arenas of action, involve different stakeholders, and suggest a variety of implementation strategies. 

### 5.1. Proactive Outreach

Pasick and Joseph have worked in multi-ethnic communities in northern California to expand reach of genetic screening. Their initial work identified a fundamental mismatch between the information provided by genetic counselors and the information that patients with low genetic literacy prefer and find meaningful [74]. They found that this mismatch stemmed from a tendency among genetic counselors to convey too much information, use complex terminology, present information in ways that were hard to follow, and engage in a one-way communication style that inhibited clients with low literacy from asking questions. In response, the researchers developed and validated a screening tool that could be administered over the phone [75] and tested strategies such as using the state-subsidized breast and cervical cancer screening program to implement the screening as an integrated service for callers to a toll-free service line. Among the callers, 59% agreed to answer questions about their family health history (FHH); 14% of these callers were at high risk of being mutation carriers, and 39% received genetic counseling versus 4% in a comparison group (68). 

Efforts like this can promote high-risk genetic services beyond specialty cancer centers and other clinical settings, where they have been concentrated and which racial-ethnic minorities and poor patients may not use. Moreover, because state breast and cervical cancer screening programs have high reach to medically underserved populations and have been developed specifically for low-income patients, this infrastructure can be capitalized on to offer HBOC screening and connect patients with resources they could access for low cost or on a sliding scale. Above all, Pasick et al.’s research shows that it is possible to engage a group of low-income minority women successfully, to complete a brief HBOC screener and seek genetic counseling when appropriate [76]. While health communication scholars have argued for decades that educational materials should be tailored to reach diverse audiences, Pasick’s work shows that such tailoring needs to be more intensive than merely translating to another language. 

Similar projects have had similar success in other settings. A safety net hospital in Phoenix, Arizona that serves predominantly low-income and uninsured patients implemented a combination of distance learning and continuing education for providers and financial assistance for patients to help them pay for screening services and diagnostic testing. Among the 84 women who were offered genetic testing, 96% agreed. This team emphasized the importance of cultural tailoring of the patient materials, and reported that Hispanic women, those with no medical insurance, and those with an FHH of breast cancer were most likely to pursue testing [77]. 

Projects such as these demonstrate how powerful it can be to partner with existing structures such as statewide cancer screening programs, patient navigator programs, community health worker networks, and other community-based organizations that have long considered fundamental causes and health inequities. And while these programs demonstrate the importance of tailoring interventions to the needs of culturally diverse groups, they also show how important it is to take services out of the clinics and bring them to the locations where these vulnerable populations actually receive care. The prospect that women of color may be as likely to pursue screening if the services are offered in geographically convenient locations and if the materials are culturally and linguistically sensitive is a compelling reminder that health inequities are not inevitable, and with the right approaches, uptake of genetic services can be similar between women of diverse backgrounds. 

### 5.2. Broadening HBOC Screening to Underserved and Minority Populations

Collecting and interpreting an individual’s family (FHH) can be one of the easiest and cheapest ways to identify red flags that might suggest a need for HBOC screening. Yet extensive research has identified numerous barriers in collecting FHH to guide patient care, both for providers [78,79,80] and for patients [81,82,83]. Several projects have sought to make the benefits of FHH screening more widely available. In 2014, the Connecticut Department of Public Health (Connecticut-DPH) analyzed data from the Behavioral Risk Factor Surveillance System (a statewide, random-digit dial survey on self-reported health habits) and recognized that in their state, low-income patients and racial/ethnic minorities were less likely to know their FHH and share it with a provider [67]. They partnered with four agencies to bring FHH education into communities: a local health department, a federally-qualified community health center, and two community organizations that provide health and social services to specific racial or ethnic minorities. Each partner developed programs that met their staffing capacities, organizational missions, and patient needs, such as mass-media campaigns and training lay health advisors to teach patients about FHH. A post-test survey showed that 85.6% of program participants agreed or strongly agreed that it is important to know one’s FHH, 75% agreed or strongly agreed that they should share their FHH with their relatives, and 82.9% agreed or strongly agreed that they should share their FHH with their healthcare provider [67]. Kaphingst et al. found similar results in partnering with a federally-qualified community health center that served low-income Latino patients. Patients who participated in an educational session run by a lay health advisor showed significant increases in self-efficacy to gather their FHH, and were more than twice as likely to understand the purpose of an FHH than those in the control group [84]. And a team of genetic services professionals at the University of Texas-Southwestern and Moncrief Cancer Institute have established a network of satellite clinics to serve patients in rural communities and trained patient navigators to help connect low-income patients to genetic services. They found substantial increases not only in uptake of genetic counseling and testing, but followed patients through to confirmatory diagnostic and treatment services and detected increases in the uptake of prophylactic mastectomy and risk-reducing salpino-oophorectomy [85,86,87,88].

These examples highlight the ways diverse partners (on the outermost ring of our framework) can broaden an intervention’s reach, using multiple implementation strategies (the inner ring of our framework). For example, both the Connecticut and Kaphingst teams found that partnering with federally-qualified community health centers was an effective way to reach patients least likely to know the benefits of FHH, and that relying on the health centers’ existing teams of lay health educators was an effective way to deliver the content. This helped to make entry to specialty healthcare services more broadly available, bringing screening for hereditary conditions out into the community, rather than sequestering them in academic medical centers. The leadership from the Connecticut-DPH was especially important in changing service sites (one of the recommended implementation science strategies) because they identified several types of agencies with different sorts of connections and resources to reach different segments of the population. These programs also showed the importance of data warehousing techniques, mass media campaigns, and preparing patients to be active participants. Finally, these examples show a path forward for developing collaborations and partnerships that might help technologically-driven interventions like telemedicine to meet their potential.

### 5.3. Aligning Policies to Make Hereditary Cancer Screening More Broadly Available

Influencing provider and patient behaviors in the clinic is important, but policy-level work is also critical to broadening the reach of genomic medicine. The Michigan health department’s Genomics Program searched their state cancer registry for women diagnosed with breast cancer before age 40 (age at diagnosis being a rough proxy for hereditary cancer syndromes) and surveyed them by mail to discover barriers in pursuing genetic testing—the second most common reason for not pursuing testing was a lack of health insurance coverage [89]. In response, the Michigan Genomics Program targeted educational outreach to medical directors of insurance companies that provide coverage to Michigan residents. They provided sample language that insurers could insert into policy certificates to align their coverage with evidence-based recommendations for HBOC screening. They began this work in 2008, before passage of the ACA, and found that only four of 24 health plans in Michigan covered HBOC screening. By 2013, (before the ACA had been fully implemented) 14 of 25 health plans had aligned their policies with expert recommendations, extending coverage for HBOC screening to 7.5 million Michigan residents [66,90,91]. 

This example shows that an expanded CFIR framework has much broader potential, and can nudge implementation science even further out of the clinic than the examples in the previous two sections. Unlike our prior examples, which were primarily focused on education and outreach that engaged patients, providers, and patient advocacy groups, this example shows how public health agencies can engage policymakers, such as insurance executives, to remove systemic barriers, encouraging adherence to evidence-based guidelines and making sure that lack of insurance does not become a barrier in accessing services. 

## 6. Conclusions

We propose an expanded CFIR framework to guide promotion of genomic medicine and broaden its reach. The expanded framework leverages the strengths of both the CFIR and fundamental cause theory. The heart of this expansion is that any effort to implement evidence-based health promotion interventions, whether genomic or otherwise, must anticipate the potential for inequities. Our expanded CFIR anticipates and seeks to offset these inequities in three ways: by broadening arenas of action, by cultivating more diverse partnerships, and coordinating activities among partners to extend over the whole healthcare arena. 

Specifically, we think implementation activities should range across a wider arena of action than implementation scientists have thus far embraced, in order to identify the ways fundamental causes might bear on the intervention. Likewise, we should be willing to foster collaborations with partners who have a deep understanding of the needs and interests of groups that are too often marginalized in our current healthcare system, including public health officials, patient advocates, social service agencies, and policymakers. Finally, we show how each of these potential collaborators can contribute via specific activities that are already part of the methods and strategies commonly found in implementation science. More cooperation among stakeholders who are attentive to health inequities could only be a good thing. 

One of the most important things to note in our integrated framework is that fundamental causes—the forces driving inequities in healthcare delivery—are multifaceted and operate in multiple arenas of action, and that they are continually in flux. It is therefore not reasonable to expect that any single intervention would be enough to mitigate inequities in healthcare delivery or health outcomes. Indeed, the examples we provide in this paper represent discrete projects, each of which targets only a portion of the problem. But by being aware of how fundamental causes operate across levels, we can recruit good partners and coordinate their activities to make a difference. Without coordinated action, we would expect a continuation and exacerbation of the inequities we are already seeing in access to HBOC screening.

In this paper, we have used HBOC screening to show how coordinated action among multiple stakeholders can mitigate inequities in access to genetic services that drive differential health outcomes. But we believe our integrated framework could potentially mitigate health inequities in other types of health outcomes as well. Finally, although we focus on the US in this paper, our framework could be applied in other countries where the healthcare system is organized and financed differently. Implementation scientists would need to identify the specific factors in the inner and outer settings that affect organization and delivery of care, and reflect on which collaborators they would need to recruit to foster change in all arenas of action. Researchers, advocates, and policymakers must consider the way power and authority is shared among institutional stakeholders in those settings to identify power dynamics that could hinder efforts to make healthcare services equitably available. But the expanded CFIR framework presents a means of analyzing the ways power shapes healthcare delivery and highlights areas of cooperative potential, which are important precursors to challenging entrenched practices that drive health inequities. 

Expecting that good intentions and passive awareness will protect genomics-informed health applications from inequitable distribution is naïve. Proactive and conceptually guided efforts will be essential to keep the past from repeating itself. To that end, we believe that an expanded CFIR framework can have a significant influence on how research is conducted, the specific outcomes it targets, and how we measure success. Imagine if each implementation study took on the following question, “If the intervention being tested in my study has the hypothesized effect, will health inequities increase or decrease?” and used it as a guide to design and execute research. The choice of contexts in which studies are set may be chosen differently with an emphasis on what inequities exist in healthcare delivery or health outcomes and whether the research will address or exacerbate those inequities. Investigative teams may design the study to concentrate on equity as a specific outcome, rather than outcomes derived as a function of who happens to be enrolled as a subject. Our expanded CFIR framework is intended to make these decisions more transparent, and recognize that the convergence of these bodies of knowledge can indeed make the best possible care the standard care for all.
